# Buffalo University Alumni Reunion

**Published:** 1902-03

**Authors:** 


### Buffalo University Alumni Reunion.

THE fourth midwinter reunion of the alumni of the medical department of the University of Buffalo was held on Washington's birthday, February 22, 1902.

The program was announced as follows: State laboratories, Daniel Lewis, M. D., New York State Commissioner of Health; Old Forts on the Niagara Frontier, Peter A. Porter, the accepted historian of Niagara; The value of wit and humor, in dealing with public questions, Herbert P. Bissell; Tri-color photography, with stereopticon, Conrad Baer.

The Gratwick research laboratory, in which is located the new cancer laboratory of the state department of health, was thrown open for inspection. The alumni invitations were accompanied by the card of the state commissioner of health, and the council and faculty of the University, inviting an inspection of the laboratory from 4 to 6 and 8 to 10 p.m. The alumni proceedings were announced to begin at 9 o'clock.

Soon after that hour, Dr. M. D. Mann, dean of the faculty, took the chair and read a telegram of regret from Dr. Daniel Lewis stating that owing to the illness and death of his mother, he would be unable to attend the meeting. After appropriate introductory remarks, Dr. Mann introduced the Hon. Peter A. Porter, of Niagara Falls, who delivered a most interesting address on the Forts of the Niagara Frontier. Mr. Porter is an enthusiastic student of the history of this region, and portrayed the stirring events relating to its occupation and early settlement in graphic English and well-chosen rhetoric.

He traced the history through the Indian, French and British eras on both sides of the river and through the American period after occupancy on this side of the Niagara, giving much interesting data and imparting instruction of great value. Such addresses inspire renewed interest in subjects of great importance.

Mr. Herbert P. Bissell took up the subject of wit and humor, especially applying it to the elaboration of public questions. He read from Artemus Ward and Mr. Dooley, giving the latter in excellent imitative dialect. Among the selections read was "Mr. Dooley on the Practice of Medicine," published in the JOURNAL, August, 1901. After an interesting exhibition of tri-color photography with stereopticon illustrations, by Conrad Baer, supper was served in the library.

### THE GRATWICK LABORATORY.

The Gratwick Laboratory, located at No. 99 High Street, opposite the Buffalo General Hospital, founded by Mrs. Martha Gratwick and her son, William H. Gratwick, was originally intended as a laboratory for general medical research and placed under control of the council of the University of Buffalo. The cancer laboratory, which is endowed by the state, was also at first under the control of the University of Buffalo, but in deference to the wishes of Governor Odell that the state should give no aid to private institutions, the laboratory was transferred to the control of the state board of health. As the Gratwick Laboratory is the only available quarters in the city for carrying on scientific research of this kind, it was decided to continue the cancer work in this building, and in order to do so the institution also had to be transferred from the control of the university council to that of the state. Under this arrangement, the state will pay for the maintenance of the institution.

In addition to the cancer research department the laboratory is completely furnished with all the latest scientific equipments for general medical-research work and will afford plenty of opportunity for independent investigation, which was the original intention of the founders. There is a pathological laboratory in charge of Dr. Harvey R. Gaylord; a bacteriological department in charge of Dr. Herman G. Matzinger and a chemical room in charge of Dr. G. H. A. Clowes. Among the other features are an animal room, in which are housed the specimens used for the development of germ cultures, two micro-photographic rooms, for obtaining negatives of the developed cultures, a combustion room for experiments with chemicals of a highly explosive nature. The building is thoroughly fire-proof, germ-proof and water-proof. Perfect ventilation is secured by means of a huge revolving fan at the top of the building, which has a draught connection with every room. This inspection proved an interesting feature of the day.

The laboratory is under the control of the state department of health, which is represented by State Commissioner Dr. Daniel Lewis, of Albany. Dr. Roswell Park, the director, is the executive head and Dr. Harvey R. Gaylord, pathologist in charge, is the scientific director of the institution.

